# Bullying Victimization and Mental Health among Migrant Children in Urban China: A Moderated Mediation Model of School Belonging and Resilience

**DOI:** 10.3390/ijerph19127135

**Published:** 2022-06-10

**Authors:** Wei Nie, Liru Gao, Kunjie Cui

**Affiliations:** 1 Institute of Urban Governance, Shenzhen University, Shenzhen 518060, China; niewei@szu.edu.cn; 2 Department of Social Work, School of Law, Nanjing University of Finance & Economics, Nanjing 210023, China; 3 Research Institute of Social Development, Southwestern University of Finance and Economics, Chengdu 611130, China; cuikunjie@swufe.edu.cn

**Keywords:** bullying victimization, migrant children, urban China, school belonging, resilience, mental health

## Abstract

School bullying victimization among children is a significant public health issue that may negatively influence their mental health. However, few studies have been conducted on the bullying of migrant children in urban China. A positive psychological perspective has rarely been adopted in examining the mechanisms through which bullying victimization influences mental health, and the protective factors remain understudied. This research investigates the factors that may contribute to reducing the negative effects of bullying victimization on mental health, focusing on the protective roles of school belonging and resilience in the association between bullying victimization and mental health. Data were collected from 1087 school-aged migrant children in Shanghai and Nanjing, China. The PROCESS macro was used to conduct moderated mediation analyses to test the hypothesized models. The results of moderated mediation modeling revealed that bullying victimization (β = −0.386, *p* < 0.001) was negatively linked with mental health through decreased school belonging (β = 0.398, *p* < 0.001). Moreover, resilience buffered the indirect negative effects of bullying victimization on migrant children’s mental health via school belonging (β = −0.460, *p* < 0.01). Specifically, lower resilience was clearly associated with stronger indirect effects. Our findings suggest that school belonging and resilience must be incorporated into mental health prevention and intervention programs targeting migrant children with bullying victimization experiences.

## 1. Introduction

Bullying is a form of aggressive behavior with the intention of harm, that takes place repeatedly over time between two persons or groups with imbalanced power [1]. It is suggested that traditional bullying includes three types: physical (such as pushing, shoving, hitting, and kicking), verbal (such as name-calling and threatening) and relational (such as spreading rumors and exclusion) [2]. School bullying is becoming an increasingly common trend worldwide and is a significant public health problem. Approximately 246 million children and adolescents suffer from school bullying worldwide every year, and approximately one-third (32%) of students in 144 countries have been victims of school bullying [3,4]. Strong evidence has shown that bullying victimization is associated with a large number of mental health problems, such as loneliness, anxiety, depression and suicidality, in children and adolescents [5,6]. The long-term negative influence of bullying victimization on mental health was verified by a five-decade longitudinal study that indicated that continual bullying victimization in childhood was linked with higher levels of mental health problems during adulthood, such as distress, depression, anxiety and suicidality [7]. Given the high prevalence and great harm of bullying victimization, it has been the focus of social work and public health research; this research has identified the path of school bullying victimization in affecting mental health and has provided effective intervention measures [8,9,10].

According to the United Nations Educational, Scientific, and Cultural Organization (UNESCO), migrant students are at high risk for suffering bullying [3]. Although the bullying victimization of migrant students and its psychological consequences have been examined extensively in Western developed countries [11,12], there are few studies on bullying victimization among migrant students in China. In China, with rapid economic development and accelerating urbanization over the past four decades, hundreds of millions of migrants have flooded into cities from their rural hometowns. Owing to the increasing flow of migrants, a large number of migrant children are emerging; migrant children are defined as children who have relocated from rural to urban areas with their parents or who were born in cities after their parents’ migration and have not obtained local household registration (hukou) at their place of destination. The latest statistics suggested that China had 34.91 million migrant children in total in 2019 [13].

The Chinese government assigns every Chinese citizen a hukou location and a rural or urban hukou type. In general, the hukou location regulates where a Chinese citizen belongs, and where he or she can be entitled to obtain benefits. For migrants without a local hukou, the current hukou system in China poses institutional barriers to access to a wide range of urban public services and welfare programs, such as school education for children, healthcare, and social welfare [14,15]. In addition, the hukou system also causes residential segregation between migrants and local urban residents, which is an important social obstacle for migrants’ urban inclusion [16]. Such institutional and social barriers have posed risks as well as significant challenges for migrant children. For example, some studies have indicated that migrant children often suffer from being devalued, rejected, excluded and discriminated against in urban areas [17,18]. Moreover, it has been found that due to their outsider identities, migrant children are more prone to being bullied than their non-migrant peers [19,20]. More recent empirical evidence has revealed that approximately two-thirds (66.6%) of migrant children experience school bullying [21]. This indicates that the likelihood of migrant children in China experiencing bullying at school is twice the likelihood at the global level (32%) reported by UNESCO [4]. Considering the adverse influences of school bullying on mental health, it is necessary to conduct further studies that focus more specifically on the underlying mechanism that may mediate or moderate the association between bullying victimization and mental health among school-aged migrant children in China.

The new and increasingly popular science of positive psychology has emphasized the value of understanding the roles of positive psychological merits and strengths (e.g., school belonging, resilience) in investigating the underlying mechanisms that improve the mental health of children and adolescents [22,23,24,25]. However, few studies have focused on protective factors that might assist children in overcoming the experience of being bullied and alleviate the harmful effect of bullying victimization on their mental health. Furthermore, a review of the literature offers no evidence concerning whether and how school belonging and resilience can mediate or moderate the relationship between bullying victimization and mental health among migrant children in China. In other words, school belonging and resilience, as two positive psychological assets, have not been examined together in the study on the association between bullying victimization and mental health. As such, based on the belongingness hypothesis and resilience theory, this study seeks to fill these gaps by exploring the underlying mechanisms of the association between school bullying victimization and mental health among migrant children in China, taking into account both school belonging and resilience.

### 1.1. School Bullying Victimization and Mental Health

Substantial evidence has indicated that bullying victimization causes short- and long-term negative influences on children’s mental health outcomes [6,26,27,28,29]. Nevertheless, this issue warrants further exploration from a new perspective. A disproportionate number of studies have been conducted from the psychopathological perspective to explore the mechanisms through which school bullying victimization affects mental health and to focus on negative psychosocial factors, such as loneliness [30,31], rumination [32], shame [33], social anxiety [34], self-stigma [35], anxiety, low self-esteem and hopelessness [31]. However, protective factors remain relatively unexplored. For example, positive psychological orientations have been found to mediate the association between bullying victimization and mental health problems [22]. Some empirical studies have shown that social support mediates the effect of bullying victimization on mental health among children [36,37] and moderates the association between bullying victimization and distress [38]. Another recent study revealed that self-compassion and hope play a mediating role in the association between bullying victimization and depression among left-behind children in rural China [39]. According to research on migrant children in China, both intrapersonal and interpersonal sources of resilience mediate the relationship between relational bullying victimization and mental health [40]. However, few studies have focused simultaneously on the mediating and moderating roles of protective factors specifically for migrant children in urban China.

### 1.2. Mediating Role of School Belonging

The belongingness hypothesis may be helpful for understanding the association between bullying victimization and children’s mental health. According to the belongingness hypothesis, the need to belong, as a fundamental human motivation, refers to the fact that “human beings have a pervasive drive to form and maintain at least a minimum quantity of lasting, positive, and significant interpersonal relationships” [41] (p. 57). Moreover, the belongingness hypothesis suggests that belonging stimulates goal-directed activity to form solid social bonds that can have a positive effect, whereas lacking belonging causes adverse reactions and negative effects on health and wellbeing [41].

School belonging refers to a student’s subjective feeling of being accepted, respected, included, and supported by peers and adults in the school environment [42]. Previous research has revealed that children’s and adolescents’ school belonging could be significantly and negatively affected by school bullying experiences [43]. Children and adolescents with bullying experiences were found to have lower levels of school belonging than those who were nonvictims and non-perpetrators [44,45,46,47]. In addition, previous studies have shown that school belonging is positively correlated with positive mental and emotional health [48,49]. Empirical evidence has indicated that school belonging can contribute to preventing anxiety and depression [50,51], boosting resilience [52], and improving self-esteem [53]. For example, an empirical study found that Chinese migrant children who reported a high level of school belonging were more prone to report a high level of mental health [54]. Similarly, a low level of school belonging may predict negative mental health outcomes [55]. Studies have indicated that school belonging buffers students against bullying victimization and the associated negative mental health outcomes [44,56,57].

However, very few studies have explored the possible mediating role of school belonging in the association between bullying victimization and mental health among children and adolescents. For example, two recent studies with students in a city in Turkey found that school belonging could reduce the adverse influence of bullying victimization on children’s internalizing and externalizing behaviors [58] and that school belonging played a mediating role in the association between school victimization and emotional problems [59]. Therefore, it is hypothesized that school belonging plays a mediating role in the association between bullying victimization and mental health. This hypothesis will be tested in this research.

### 1.3. Moderating Role of Individual Resilience

Resilience can be conceptualized as “positive adaptation, or the ability to maintain or regain mental health despite experiencing adversity” [60]. Resilience is also defined as the ability to adapt or respond flexibly and resourcefully to a number of social situations with various demands [61]. A meta-analysis indicated that resilience was positively associated with positive indicators of mental health (such as positive affect and life satisfaction) and negatively associated with negative indicators of mental health (such as depression and anxiety) [62]. Previous studies have suggested that students with a high level of resilience are less likely to be bullied [63,64,65,66,67]. Moreover, some bullying victims can overcome the negative effects of victimization through their own resilience [68]. Therefore, promoting resilience has been suggested as one way of decreasing the adverse influence of victimization [69]. Furthermore, it has been confirmed that resilience is a protective factor for mitigating the impact of being bullied on victims’ mental health [63,64]. More recently, one study found that resilience moderated the relation between peer victimization and depressive symptoms among migrant children in urban China [18]. Another cross-sectional study of 1903 primary school students in China indicated that the moderating effect of resilience on the association between bullying victimization and social anxiety was statistically significant only in girls [70]. As depressive symptoms and anxiety are recognized as negative indicators of mental health, it is reasonable to speculate that resilience can serve as a moderator to buffer the negative influence of bullying victimization on mental health. Nevertheless, to the best of our knowledge, studies on the possible moderating role of resilience between bullying victimization and mental health in migrant children are quite scarce.

### 1.4. The Context of This Study

**Figure 1:**
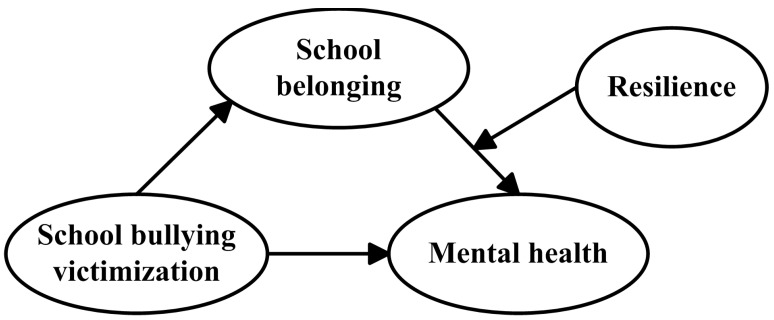
A conceptual diagram of the hypothesized relations in a moderated mediation model.

There has been little research on bullying victimization among migrant children in contemporary urban China. Moreover, previous research has focused mostly on negative psychological processes in exploring the mechanism through which bullying victimization affects mental health. Despite limited evidence of the effects of protective factors (such as school belonging and resilience) on the association between bullying victimization and children’s mental health, researchers have rarely integrated school belonging and resilience when examining school belonging, and resilience may mediate or moderate the relationship. Therefore, inspired by the belongingness hypothesis and resilience theory, this study aims to explore the positive and protective roles of school belonging and resilience in the correlation between school bullying victimization and mental health among migrant children in urban China. We expect that school belonging will mediate the influence of bullying victimization on mental health and that resilience will play a moderating role (see Figure 1). Thus, we formulate two hypotheses as follows:

**Hypothesis** **1.** *School belonging mediates the association between bullying victimization and mental health among migrant children*.

**Hypothesis** **2.** *Resilience moderates the mediating effect of school belonging on the association between bullying victimization and mental health among migrant children*.

## 2. Methods

### 2.1. Participants

The participants were pupils of grades 5 and 6 from 6 schools with high percentages of migrant children in Shanghai and Nanjing, China. Shanghai and Nanjing are the center and subcenter, respectively, of the economic zone of the Yangtze River Delta, which attracts large numbers of migrants from across China seeking higher-paying jobs. Thus, it is appropriate to collect data from a diverse variety of migrant children in both cities. 

Multistage stratified random sampling was used to select 408 and 689 migrant children from Shanghai and Nanjing, respectively. First, three districts with high concentrations of migrants were selected from each city. Second, a primary school was randomly selected from the list of the migrant children’s schools in each district. Third, students in grades 5–6 were selected by cluster sampling from each school. Following the above sampling procedure, the initial sample consisted of 1220 children in total (i.e., 1087 migrant children and 133 non-migrant children). According to the subject of this study, all non-migrant children were excluded. Ultimately, only the 1087 migrant children were included in the study.

### 2.2. Measures

#### 2.2.1. Dependent Variable

The dependent variable was mental health. This study used the 5-point Likert scale of mental health from the 2013–2014 China Education Panel Survey (CEPS), which was a national longitudinal survey of children [54]. The scale was composed of five items that inquired about the occurrence of negative psychological conditions in the past seven days. Examples of items were depression, anxiety, unhappiness, meaninglessness, and sadness. Responses included never (1), rarely (2), sometimes (3), most of the time (4), and always (5). After the scores of the five items were reversed, a continuous variable for mental health was generated by summing all the items. The scores could range from 5 to 25, with higher scores indicating better mental health (Cronbach’s α = 0.891). Confirmatory factor analysis (CFA) of the present data confirmed the good construct validity of the scale (χ^2^ = 22.413, df = 5, RMSEA = 0.057, TLI = 0.988, CFI = 0.994).

#### 2.2.2. Independent Variable

We used the bullying victimization scale from the 2015 and 2018 Program for International Student Assessment (PISA) surveys, which were organized by the Organization for Economic Co-operation and Development (OECD) [71]. The scale was composed of six items that inquired about the occurrence of physical, verbal, and relational bullying events [71,72]. Six items asked the participants about the frequency with which they had experienced bullying events as victims in school during the past 12 months: (1) Other students exclude me from group activities on purpose (relational bullying); (2) Other students make fun of me (verbal bullying); (3) Other students threaten me (verbal bullying); (4) Other students deliberately take away or destroy my things (physical bullying); (5) Other students hit or push me (physical bullying); and (6) Other students spread nasty rumors about me (relational bullying). Responses included never or almost never (1), a few times a year (2), a few times a month (3), and once a week or more (4). A continuous variable for bullying victimization was generated by summing all the items. The scores could range from 6 to 24, with a higher score indicating a higher level of bullying victimization (Cronbach’s α = 0.858). The CFA of the scale created a saturated model that had high factor loadings ranging from 0.679 to 0.774.

#### 2.2.3. Mediating Variable

In this research, we hypothesized that school belonging would play a mediating role between bullying victimization and mental health. School belonging was measured by the scale from PISA [73], which was composed of six items. The participants were asked whether they agreed with the six following positive statements about their school life: (1) being an outsider; (2) easily making friends; (3) feeling a sense of belonging at school; (4) feeling awkward or out of place at school; (5) feeling liked by other students; and (6) feeling lonely at school. Responses included strongly agree (1), agree (2), disagree (3), and strongly disagree (4). After the reversed items were recoded (i.e., 1, 4, 6), a continuous variable for school belonging was generated by summing all the items. The scores could range from 6 to 24, with a higher score indicating a higher level of school belonging (Cronbach’s α = 0.781).

#### 2.2.4. Moderating Variable

In this study, we hypothesized that resilience would play a moderating role in the association between school belonging and mental health. Resilience was measured using the 25-item Connor-Davidson Resilience Scale (CD-RISC), which is a self-administered scale developed to measure the reaction to a particular situation (e.g., change, stressor, and challenge). Examples of items include the following “able to adapt to change” and “tend to bounce back after illness or hardship” [74]. Our survey asked the participants to rate each item with reference to the previous month based on a 5-point Likert scale (0 = never, 1 = rarely, 2 = sometimes, 3 = often, 4 = almost always). A continuous variable for resilience was generated by summing all the items. The scores could range from 0 to 100, with a higher score indicating a higher level of resilience (Cronbach’s α = 0.924). Previous research has confirmed that the Chinese version of the scale has high internal reliability and convergent validity among Chinese adolescents [75].

#### 2.2.5. Control Variables

In addition to bullying victimization, the following variables that were likely to be connected to children’s mental health were controlled based on the literature [10,54,76,77]: (1) gender (0 = female, 1 = male); (2) age; (3) hukou (0 = urban, 1 = rural); (4) geographic location (0 = Shanghai, 1 = Nanjing); (5) only child status (0 = no and 1 = yes); (6) family’s economic condition (1 = very poor, 2 = poor, 3 = average, 4 = rich, 5 = very rich); (7) school type (0 = private school, 1=public school); (8) number of school changes; and (9) academic performance (1 = not good, 2 = below average, 3 = average, 4 = above average, 5 = good). Table 1 reports descriptive statistics of the selected variables used in the analyses.

### 2.3. Data Analysis

First, Pearson correlation analyses were carried out to explore the correlations among the main variables of bullying victimization, school belonging, resilience and mental health. Second, multiple linear regression analyses were conducted to examine the main effect of bullying victimization on mental health. On the basis of a model that included only control variables, bullying victimization was added into the model as an independent variable. Third, a simple mediation analysis proposed by Hayes [78] was carried out to explore the mediating effect of school belonging on the association between bullying victimization and mental health. Fourth, a moderated mediation analysis (model 14) proposed by Hayes was carried out to explore whether resilience moderated the indirect effect of bullying victimization on mental health via school belonging. Specifically, mediation and moderated mediation analyses were conducted using models 4 and 14, respectively, in the PROCESS macro for SPSS developed by Hayes [78]. A total of 5000 bootstrapping samples were used to determine the significance of the mediation and moderated mediation effects with 95% confidence intervals (CIs). If the CI did not include zero, the effect was significant. The data were analyzed using IBM SPSS Statistics version 21.0 (IBM, Armonk, NY, USA) and Hayes’ SPSS PROCESS 3.4 macro [79].

## 3. Results

### 3.1. Correlation Analysis

Intercorrelations among the studied variables are presented in Table 2. As hypothesized, bullying victimization was significantly and negatively associated with mental health (γ = −0.421, *p* < 0.01) and school belonging (γ = −0.461, *p* < 0.01). School belonging was significantly and positively associated with mental health (γ = 0.534, *p* < 0.01). Resilience was significantly and positively correlated with mental health (γ = 0.417, *p* < 0.01). In addition, bullying victimization was significantly and negatively correlated with resilience (γ = −0.223, *p* < 0.01). School belonging was significantly and positively correlated with resilience (γ = 0.530, *p* < 0.01).

### 3.2. Main Effect of Bullying Victimization

The results of the main effect model analysis are displayed in Table 3. Only control variables were included in model 1. On the basis of model 1, the independent variable of bullying victimization was added to model 2. Bullying victimization was significantly and negatively correlated with mental health (b = −0.509, *p* < 0.001). Additionally, bullying victimization had the strongest correlation with mental health among all the control variables and independent variables (β = −0.386). The control variables accounted for 8.9% of the variance in mental health, but the R-squared increased to 23% when the independent variable of bullying victimization was added to model 2, as seen in Table 3. This showed that bullying victimization accounted for 14.1% of the variance in mental health, denoting a relatively high explanatory power.

### 3.3. Mediating Effect of School Belonging

The results of the mediation model are indicated in Table 4. Bullying victimization was significantly and negatively associated with migrant children’s school belonging (b = −0.414, *p* < 0.001) when other variables were controlled. Meanwhile, bullying victimization was the most strongly correlated compared to all the control variables (β = −0.407) (model 3). This correlation indicated that a high degree of bullying victimization corresponded to a low level of school belonging. Then, bullying victimization and school belonging were added to model 4. Bullying victimization had a significant negative effect on children’s mental health (b = −0.295, *p* < 0.001). School belonging significantly and positively predicted children’s mental health (b = 0.516, *p* < 0.001). School belonging had the strongest correlation of all the variables (β = 0.398), as seen in model 4. In addition, Table 4 shows that the control variables and bullying victimization explained 30.6% of the variance in school belonging. The R-squared increased to 34% when the independent variable of bullying victimization and the mediating variable were added to model 4. The R-squared increased by 11% compared to model 2 in Table 3. This finding suggests that school belonging explained 11% of the variance in mental health, indicating a relatively high explanatory power.

Mediation analysis using a bias-corrected bootstrap method was conducted to examine the mediating roles of school belonging between bullying victimization and mental health. The results are displayed in Table 5. Bullying victimization had a significant direct effect on mental health (effect = −0.295, 95% bootstrap CI range from −0.368 to −0.222). Direct effects accounted for 58% of the total effect. School belonging significantly mediated the relationship between bullying victimization and mental health (effect = −0.2135, 95% bootstrap CI range from −0.2653 to −0.1674). The indirect effect of school belonging explained 42% of the total effect of bullying on children’s mental health. The findings that school belonging was an essential source in reducing mental health risks in the face of school bullying experiences supported the mediation analyses.

### 3.4. Moderating Effect of Resilience

Table 6 indicates the results of the moderated mediation analyses treating school belonging as the mediator when resilience was used as the moderator in the association between school belonging and mental health. In model 6, the main effect of resilience on children’s mental health was significant (*p* < 0.001). Resilience was positively associated with the level of mental health (b = 0.136, β = 0.474, *p* < 0.001), indicating that a high level of resilience was predicted to enhance the mental health outcome of migrant children. The interaction between school belonging and resilience was significantly and negatively correlated with migrant children’s mental health (b = −0.004, β = −0.460, *p* < 0.01). The moderated mediation model explained 36.9% of the variance in mental health, denoting a relatively high explanatory power. Therefore, these findings show that the mediating effect of school belonging on the correlation between bullying victimization and mental health was moderated by resilience.

**Figure 2:**
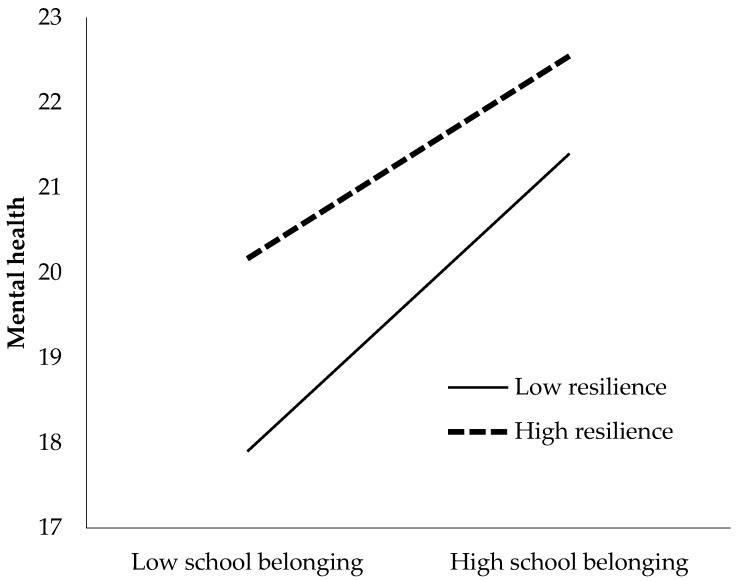
Moderating effect of resilience on the correlation between bullying victimization and mental health.

The correlation between school belonging and mental health was plotted when the levels of resilience were 1 SD below and 1 SD above the mean. This represents the simple effect of bullying victimization at two levels of resilience (as a moderator) [80]. Figure 2 depicts school belonging being moderated by resilience.

As indicated in Table 7, the mediating effect of school belonging changed according to the level of resilience. The conditional indirect effect of school belonging on mental health for low (effect = −0.192, SE = 0.0264, 95% boot CI range from −0.2447 to −0.1406), medium (effect = −0.1614, SE = 0.0229, 95% boot CI range from −0.2085 to −0.1178) and high levels of resilience (effect = −0.1308, SE = 0.0258, 95% boot CI range from −0.1841 to −0.0835) were significant. These results indicated that the indirect effect was the strongest when resilience was low (M − 1SD).

The bias-corrected 95% confidence interval for the index of moderated mediation was from 0.0003 to 0.0032 (Table 8), which did not contain zero. This further showed that the indirect effects of bullying victimization on mental health via school belonging were significantly moderated by resilience.

## 4. Discussion

With a sample of 1087 migrant children recruited from Shanghai and Nanjing in eastern China, this study examined the mediating effects of school belonging on the relationship between bullying victimization and mental health among migrant children and the moderating role of resilience in the mediating effects. To the best of our knowledge, this is the first study to examine the mediating role of school belonging and the moderating role of resilience in the association between bullying victimization and mental health among migrant children in China. The major findings and their implications for social work interventions and social policy are discussed as follows.

Consistent with the previous research [21], the findings of this study indicated that 50.7% of migrant children in China experienced at least one type of school bullying, which is 18.7 percentage points higher than the global level (32%) for children reported by UNESCO [4]. The findings of this research also displayed that 26.6% of migrant children in China frequently (at least a few times a month) experienced at least one type of school bullying. Similarly, it was suggested that 28.8% of migrant children in the United States frequently experienced at least one form of bullying [11]. Thus it verified that migrant children are more likely to experience bullying victimization. Furthermore, the findings of this study also showed that 23.8% of migrant children in China most often or always experienced emotional disorders, such as depressive and anxiety disorders. This is significantly higher than the global level (10–20%) for children and adolescents [81,82]. Therefore, it is confirmed that migrant children are more likely to be at greater risk of mental health problems.

The results confirmed the mediating role of school belonging between bullying victimization and mental health among migrant children in China, which supports Hypothesis 1. This evidence implies that migrant children who experience bullying victimization tend to report a lower level of school belonging and be more prone to manifesting mental health problems. These findings are consistent with empirical results showing that school belonging can mediate the effect of bullying victimization on mental health outcomes [58,59]. Bullying victimization can produce immediate and delayed negative effects on children’s ability to adjust to school [83,84]. Thus, when migrant students experience more bullying victimization, they find it difficult to establish good interpersonal relationships with peer groups, and identify with peer groups and they are more prone to regard school as an unhappy and unsafe environment, which in turn reduces their levels of school belonging. The findings are consistent with previous research that showed that children’s school belonging could be significantly and negatively affected by school bullying experiences [43]. On the other hand, according to the belongingness hypothesis, a sense of belonging is a key factor contributing to sustaining mental health [41]. Lacking school belonging could result in mental health problems such as loneliness, sadness, depression and anxiety.

Moreover, this study found that the mediating effect of school belonging was moderated by resilience, with the effect being more potent for migrant children with a low level of resilience. That is, the mediating effects varied when resilience decreased, especially among migrant children with a low level of resilience. Thus, Hypothesis 2 was confirmed. The findings differ from those of previous studies that showed that resilience served as a mediator linking bullying victimization and mental health (such as the negative indicators of mental health, depression, self-harm) [23,24]. However, the results are consistent with those of earlier studies indicating the moderating effect of resilience on the association between bullying victimization and mental health [18,70]. In fact, three models of resilience theory—compensatory, protective, and challenge—can be used to interpret how resilience operates to buffer the negative effects of risks [85,86]. Specifically, the findings confirm the protective model of resilience, which indicates that resilience, as a protective factor, operates interactively to moderate the adverse effects of risks by means of counteracting and obtaining assets or resources [87]. For migrant children, resilience can help them effectively cope with low levels of school belonging resulting from bullying victimization and mitigate the impact of victimization experiences on mental health. Migrant children with high levels of resilience have benefits in obtaining important assets or resources (such as self-efficacy, family support, and social support) to counter low levels of school belonging caused by bullying victimization [24]; thus, the adverse effect on mental health can be reduced.

However, several limitations of this study should be noted. First, we employed a cross-sectional data, which could not be used to determine any causalities among the research variables. Hence, there is a need to conduct longitudinal studies in the future to pinpoint possible cause–effect relationships. Second, we collected the data in two large cities in the Yangtze River Delta in China, which could restrict the generalizability of our findings across China. Future research based on nationally representative data will need to be conducted to extend the results to Chinese migrant students from other regions. Third, the independent, dependent, moderating and mediating variables analyzed in this research were collected via the participants’ self-reports, which may add the risk of statistical inaccuracy. Therefore, mixed source data should be adopted, in which there are not only behavioral or performance variables, but also other variables measured by the reports of migrant children and their teachers, parents, and peers. Finally, some influencing factors on mental health were not included as control variables in this study. Future research should include additional important control variables, such as physical activity.

## 5. Conclusions

Although numerous studies have established the harmful effect of school bullying victimization on children’s mental health, it is uncertain what mediating mechanism may explain this negative association and what moderating mechanism may buffer this negative association among Chinese migrant children. The present study finds that school bullying victimization damages migrant children’s mental health by reducing their sense of school belonging. Furthermore, resilience buffers the indirect negative effects of bullying victimization on migrant children’s mental health via school belonging.

This study contributes to the literature on the correlation between bullying victimization and mental health among migrant children. It also provides first-hand empirical evidence on the protective role of school belonging and resilience in the link between bullying victimization and mental health among migrant children in urban China, which further deepens and expands our understanding of how to improve mental health from the perspectives of the belongingness hypothesis, resilience theory and positive psychology.

Last, this study has significant implications for the development of bullying victimization prevention and intervention. The findings also suggest some promising directions for social work practices. The remarkable and strong effects of school belonging and resilience imply that it is necessary to develop interventions focused on both school belonging and resilience for migrant children at risk of being bullied because the use of such interventions can be an effective strategy to prevent them from experiencing mental health problems. School belonging could be successfully improved by building students’ strengths and improving positive interactions between students and teachers and among students [88]. Resilience could be improved by adopting a whole-child approach [65] and focusing on developing programs that could build migrant children’s strength and enhance support from peers, teachers and parents. School-based lectures, consultations and workshops could be conducted to cultivate migrant children’s sense of optimism and self-efficacy and help them build their strength. Furthermore, activities that help children receive social skills training, develop problem-solving skills and set goals individually or in groups could improve the peer relationships and mental health of children [89,90]. Moreover, interventions targeting children’s emotional regulation and coregulation with teachers and peers have also been found to be effective in improving their school belonging and psychological wellbeing [91]. Considering migrant parents’ limited time, ability and resources, it is important to conduct online and offline family education services that could teach parents how to foster good parent–child relationships and how to give their children appropriate emotional, informational, appraisal and instrumental support.

## Figures and Tables

**Table 1:** Descriptive statistics of selected variables in the analyses.

	*N* (%)	Mean (SD)	Range
Gender			
female	473 (43.5)		
male	614 (56.5)		
Hukou			
urban	240 (22.1)		
rural	847 (77.9)		
Geographic location			
Shanghai	408 (37.5)		
Nanjing	679 (62.5)		
Only child status			
no	887 (81.6)		
yes	200 (18.4)		
School type			
private	854 (78.6)		
public	233 (21.4)		
Number of school changes			
0	719 (66.1)		
1	210 (19.3)		
2	81 (7.5)		
3	64 (5.9)		
4	12 (1.1)		
5	1 (0.1)		
Age		12.3 (0.8)	10–17
Family’s economic condition		3.1 (0.6)	1–5
Academic performance		3.3 (1.2)	1–5
Bullying victimization		8.4 (3.7)	6–24
School belonging		19.8 (3.8)	6–24
Resilience		72.8 (17.0)	0–100
Mental health		20.3 (4.9)	5–25
N	1087	1087	

**Table 2:** Correlation matrix of the study variables.

	1	2	3	4
1. Bullying victimization	1			
2. School belonging	−0.461 **	1		
3. Resilience	−0.223 **	0.530 **	1	
4. Mental health	−0.421 **	0.534 **	0.417 **	1

** *p* < 0.01.

**Table 3:** Results of the multiple linear regression model.

	Model 1 Mental Health			Model 2 Mental Health		
	b	S.E.	β	b	S.E.	β
Male	0.401	0.290	0.041	0.674 *	0.268	0.069
Age	−0.073	0.199	−0.011	−0.128	0.183	−0.020
Rural hukou	0.098	0.348	0.008	0.138	0.320	0.012
Only child status	0.345	0.373	0.027	0.261	0.343	0.021
Nanjing	1.912 ***	0.341	0.190	1.194 ***	0.318	0.119
Number of school changes	−0.112	0.158	−0.022	−0.025	0.146	−0.005
Public school	−2.024 ***	0.394	−0.170	−1.780 ***	0.363	−0.149
Family’s economic condition	1.045 ***	0.251	0.125	0.915 ***	0.231	0.109
Academic performance	0.658 ***	0.127	0.155	0.445 ***	0.118	0.105
Bullying victimization				−0.509 ***	0.036	−0.386
Intercept	14.780	2.677		21.014 ***	2.470	
R^2^	0.089			0.230		
F	11.743 ***			32.145 ***		

Unstandardized (b) and standardized (β) coefficients are reported. * *p* < 0.05. *** *p* < 0.001.

**Table 4:** Results of mediation model.

	Model 3 School Belonging			Model 4 Mental Health		
	b	S.E.	β	b	S.E.	β
Male	0.252	0.196	0.033	0.545 *	0.248	0.055
Age	−0.146	0.1343	−0.029	−0.052	0.170	−0.008
Rural hukou	−0.297	0.234	−0.033	0.291	0.297	0.025
Only child status	0.056	0.252	0.006	0.232	0.318	0.018
Nanjing	0.693 **	0.233	0.089	0.837 **	0.296	0.083
Number of schol changes	−0.159	0.107	−0.040	0.057	0.135	0.011
Public school	−0.930 ***	0.266	−0.101	−1.300 ***	0.338	−0.109
Family’s economic condition	0.728 ***	0.169	0.112	0.539 *	0.216	0.064
Academic performance	0.747 ***	0.086	0.228	0.060	0.112	0.014
Bullying victimization	−0.414 ***	0.027	−0.407	−0.295 ***	0.037	−0.224
School belonging				0.516 ***	0.039	0.398
Intercept	20.235 ***	1.834		10.576 ***	2.445	
R^2^	0.306			0.340		
F	47.454 ***			50.350 ***		

Unstandardized (b) and standardized (β) coefficients are reported. * *p* < 0.05. ** *p* < 0.01. *** *p* < 0.001.

**Table 5:** Results of the mediation analysis.

	Effect	BootSE	BootLLCI	BootULCI	Effect versus Total Effect
Direct effect (Bullying victimization → Mental health)	−0.2950 ***	0.0372	−0.3680	−0.2220	58.0%
Indirect effect (Bullying victimization → School belonging → Mental health)	−0.2135 ***	0.0249	−0.2653	−0.1674	42.0%
Total effect	−0.5085 ***	0.0363	−0.57973	−0.4373	

*** *p* < 0.001.

**Table 6:** Results of moderated mediation analysis.

	**Model 5 School Belonging**			**Model 6 Mental Health**		
	b	S.E.	β	b	S.E.	β
Male	0.252	0.196	0.033	0.490 *	0.244	0.050
Age	−0.146	0.134	−0.029	−0.080	0.166	−0.012
Rural hukou	−0.297	0.234	−0.033	0.444	0.291	0.038
Only child status	0.056	0.252	0.006	0.212	0.311	0.017
Nanjing	0.693 **	0.233	0.089	0.534 *	0.295	0.053
Number of school changes	−0.159	0.107	−0.040	0.100	0.132	0.019
Public school	−0.930 ***	0.266	−0.101	1.351 ***	0.331	−0.113
Family’s economic condition	0.728 ***	0.169	0.112	0.454 *	0.212	0.054
Academic performance	0.747 ***	0.086	0.228	−0.082	0.113	−0.019
Bullying victimization	−0.414 ***	0.027	−0.407	−0.309 ***	0.036	−0.235
School belonging				0.707 ***	0.120	0.546
Resilience				0.136 ***	0.031	0.474
School belonging × Resilience				−0.004 **	0.002	−0.460
Intercept	20.235 ***	1.834		4.578	3.130	
R^2^	0.306			0.369		
F	47.454 ***			48.159 ***		

Unstandardized (b) and standardized (β) coefficients are reported. * *p* < 0.05. ** *p* < 0.01. *** *p* < 0.001.

**Table 7:** Conditional indirect effect at specific levels of the moderator when school belonging was treated as a mediator.

Moderator: Resilience	Effect	BootSE	BootLLCI	BootULCI
Low resilience (M − 1SD)	−0.1920	0.0264	−0.2447	−0.1406
Medium resilience (M)	−0.1614	0.0229	−0.2085	−0.1178
High resilience (M + 1SD)	−0.1308	0.0258	−0.1841	−0.0835

**Table 8:** Index of moderated mediation.

	Index	BootSE	BootLLCI	BootULCI
Resilience	0.0018	0.0007	0.0003	0.0032

## Data Availability

The datasets generated and/or analyzed during the current study are not publicly available but are available from the corresponding author on reasonable request.
